# National Wastewater Surveillance of Illicit Tobacco and Vaping Use Trends in Australia

**DOI:** 10.1001/jamanetworkopen.2025.57319

**Published:** 2026-02-09

**Authors:** Zhe Wang, Qiuda Zheng, Phong K. Thai, Coral Gartner, Jake W. O’Brien, Richard Bade, Rory Verhagen, Wayne Hall, Daniel Stjepanović, Bradley S. Simpson, Emma L. Keller, Kevin V. Thomas, Jochen F. Mueller, Ben Tscharke

**Affiliations:** 1 Queensland Alliance for Environmental Health Sciences (QAEHS), The University of Queensland, Woolloongabba, Queensland, Australia; 2 NHMRC Centre of Research Excellence on Achieving the Tobacco Endgame, School of Public Health, The University of Queensland, Herston, Queensland, Australia; 3 National Centre for Youth Substance Use Research (NCYSUR), School of Psychology, The University of Queensland, St Lucia, Queensland, Australia; 4 Clinical and Health Sciences, University of South Australia, Adelaide, South Australia, Australia

## Abstract

**Importance:**

Australia is leading the world in efforts to reduce tobacco use by implementing high cigarette taxes and restrictive regulations on nicotine vaping products. However, concerns have emerged that these policies may unintentionally drive the expansion of illicit tobacco and vaping markets, potentially undermining public health gains.

**Objectives:**

To assess spatial and temporal changes in total nicotine, tobacco-derived nicotine, and illicit tobacco use across Australian regions of different remoteness from 2017 to 2023.

**Design, Setting, and Participants:**

This longitudinal, cross-sectional wastewater study was performed from April 2017 to April 2023. Wastewater samples were collected from as many as 55 wastewater treatment plants (WWTPs) in Australia, including 3 remoteness levels: major cities, inner regional, and outer regional to remote areas. The selected WWTPs serve more than 50% of the Australian population.

**Main Outcomes and Measures:**

Nicotine metabolites (cotinine and hydroxycotinine) and the tobacco-specific alkaloid (anabasine) were analyzed in wastewater samples using a validated liquid-chromatography tandem mass spectrometry method. Total nicotine and tobacco-derived nicotine consumption were back-estimated. Illicit tobacco use was identified in combination with the tobacco sales data.

**Results:**

Wastewater samples collected across Australia, representing 14 million people, were analyzed for back-estimation. Total nicotine consumption declined fastest in outer regional to remote areas (−2.2% annually; 95% CI, −3.2% to −1.1%), followed by inner regional areas (−1.4% annually; 95% CI, −2.1% to −0.8%), and remained stable in major cities. By comparison, tobacco-derived nicotine consumption decreased faster in major cities (−5.0% annually; 95% CI, −8.3% to −1.9%) and inner regional areas (−9.8% annually; 95% CI, −12.5% to −7.3%) than in the outer regional to remote areas (−2.3% annually; 95% CI, −6.0% to 1.8%). Illicit tobacco use was estimated to have increased from 1350 to 3400 tons from 2017 to 2023.

**Conclusions and Relevance:**

In this cross-sectional study of wastewater surveillance in Australia, different trends of tobacco use were observed across regions, accompanied by increasing use of illicit tobacco and vaping products. These findings provide evidence for future tobacco and vaping control policies. Ongoing wastewater monitoring is essential for evaluating new tobacco and vaping product control measures implemented in 2024.

## Key Points

**Question:**

Following recent tobacco control policies, what are the trends in smoking and vaping in Australia?

**Findings:**

In this cross-sectional study, with wastewater samples representing up to 50% of the Australian population, a continued decline in tobacco use was observed, particularly in regional areas. However, the results also suggested a recent increase in nicotine consumption primarily sourced from vaping products as well as an expansion of the illicit tobacco market in Australia.

**Meaning:**

This study found that tobacco use has decreased in Australia; however, the rise in illicit tobacco and vaping underscores the need for additional measures to maintain progress in tobacco control.

## Teaser

This cross-sectional study uses wastewater samples to estimate the spatial and temporal trends in nicotine use, including vaping, from 2017 to 2023 in Australia.

## Introduction

Smoking remains a leading risk factor for premature mortality and morbidity in Australia, accounting for 7.6% of the total disease burden and 11.6% of all deaths in 2024.^1^ This persists despite significant government efforts to reduce tobacco smoking, notably through high tobacco taxes that have increased annually, one of the most effective policies in reducing smoking prevalence.^2^ Australia has no domestic tobacco manufacturing, so all tobacco sold in Australia is imported. This means consumption trends are particularly sensitive to the balance between taxation policies driving down consumption or causing diversion to illicit markets. In 2010, a 25% one-off tax increase was implemented, followed by annual 12.5% increases from 2013 to 2020, and 3 additional 5.0% annual increases from 2023 to 2025.^3^

As a result of these changes, the daily smoking rate in Australia declined markedly from 15.0% in 2010 to 8.3% in 2022 to 2023.^4^ This progress surpassed the goal of reducing daily smoking to 10.0% by 2025, bringing Australia closer to the goal of a 5.0% daily smoking prevalence by 2030.^5^ However, high rates of taxation appear to have led to the growth of substantial illicit markets in tobacco and e-cigarettes, or vaping products.^6,7^

The growing use of vaping products has raised public health concerns, prompting governments to implement policies to limit their availability.^8^ Nicotine-containing vaping products have never been legally available for general consumer use in Australia, yet their use has surged. From 2016 to 2023, daily vaping prevalence increased from 0.5% to 3.5%.^7^ The highest rates have been observed among young adults, largely driven by easy availability and an abundance of appealing flavors.^9^ In response, regulations have been introduced to limit youth access while permitting adults to access vaping products via prescription for smoking cessation purposes.^8,10,11^

To effectively monitor the impact of tobacco control policies on tobacco smoking and nicotine vaping, it is essential to understand their use among different sociodemographic groups.^12^ Wastewater analysis has proven to be an effective tool for obtaining this information^13,14^ when used appropriately.^15^ It is possible to use it to monitor trends in both total nicotine and tobacco-derived nicotine consumption.^16,17,18,19^ This approach also enables comparison of consumption across geographic regions.^20,21^ By integrating wastewater and sales data, it is possible to estimate the proportion of tobacco use that originates from illicit sources.

This study aimed to investigate the temporal trends of total nicotine and tobacco-derived nicotine consumption across different remoteness regions of Australia by monitoring nicotine metabolites (cotinine and hydroxycotinine) and anabasine in wastewater. More importantly, by combining the wastewater-based estimate with annual tobacco sales data, the study sought to assess the trajectory of illicit tobacco use.

## Methods

### Data Source

This longitudinal, cross-sectional study adhered to the Strengthening the Reporting of Observational Studies in Epidemiology (STROBE) reporting guideline for cross-section studies.^22^ This project has been reviewed by UQ Research Ethics and Integrity and was deemed to be exempt from ethics review under the National Statement on Ethical Conduct in Human Research and relevant University of Queensland policy.

### Sample Collection

The wastewater samples analyzed in this study were collected by the Australian National Wastewater Drug Monitoring Program (NWDMP) from April 2017 to April 2023,^23^ which were collected from areas covering more than 50% of the Australian population.^20^ Details of sampling, catchment population, and flow data collection are provided in eTables 1 and 2 in Supplement 1.

### Data Analysis

Two nicotine biomarkers (cotinine and hydroxycotinine) and a tobacco-specific alkaloid (anabasine) were analyzed in wastewater samples using validated liquid-chromatography tandem mass spectrometry methods.^24^ Back-estimations were performed, with a flowchart (eFigure 1 in Supplement 1) illustrating the interrelationships among equations. Definitions of all terms in the equations are provided in eFigure 2 in Supplement 1. First, total nicotine consumption was calculated from nicotine metabolites (Equation 1): 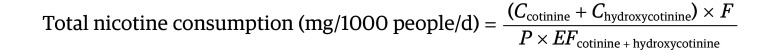
Then, tobacco use was estimated from anabasine to units of cigarettes (Equation 2): 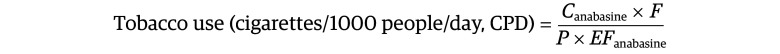
To convert the anabasine tobacco–specific measurements to the equivalent consumption estimate of tobacco-derived nicotine, tobacco use and the milligrams of nicotine per cigarette are multiplied (Equation 3).
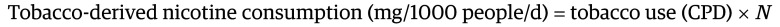
*C* represents the concentration of biomarkers (in micrograms per liter), *F* represents the flow of the wastewater during the sampling period (in megaliters per day), and *P* represents the population covered by the wastewater catchment (×1000). *EF*_cotinine + hydroxycotinine_ represents the excretion rate of nicotine biomarkers after nicotine consumption (74%).^13^ *EF*_anabasine_ is 1.13 µg/cigarette,^19^ and *N* is nicotine dose of each cigarette (0.9 mg/cigarette).^25^

The sampling sites were classified into 3 remoteness levels. The Australian Bureau of Statistics categorizes areas based on relative access to services as very remote, remote, outer regional, inner regional, and major cities.^26^ Since there were only 2 sites in the remote category and none in the very remote category, these were combined with the outer regional category and labeled as outer regional to remote. As a result, the sites were ultimately categorized into 3 remoteness levels: major cities, inner regional, and outer regional to remote.

Both the national weighted average total nicotine consumption and tobacco-derived nicotine consumption from each month were calculated by combining mean values of nicotine consumption in different remoteness areas, with the proportion of Australians living in different areas. That is, 72% of all Australian residents live in in major cities, 18% in inner regional areas, and the other 10% in outer regional to remote areas. The weighted average tobacco use of each month was calculated (Equation 4)^27^:
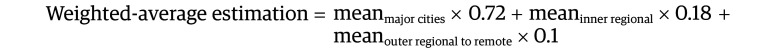
Then, wastewater-based estimates (WBE) of annual tobacco use were calculated (Equation 5), where tobacco content is assumed to be 0.7 g/cigarette on average.
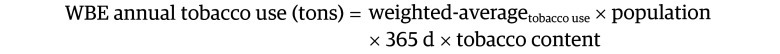
The tobacco industry–funded report by FTI Consulting^28^ on illicit tobacco in Australia 2024 included (1) national legal tobacco use data (FTI legal estimates): legal sales data in Australia and estimates of products brought in by international arrivals based on source country; and (2) national estimates of illicit tobacco use (FTI illicit estimates) in Australia over the period from 2013 to 2023.^28^ Furthermore, the FTI report also presents police seizures data, which includes seizure data from both Australian Border Force and the Australian Taxation Office.^28^

The WBE illicit tobacco use was calculated in Equation 6:
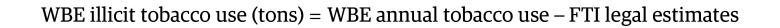
The legal tobacco use estimates (sourced from the report, FTI legal estimates) were subtracted from the annual tobacco use in wastewater estimates (WBE annual tobacco use).

### Statistical Analysis

Data were plotted by grouping each site by remoteness categories by time. Temporal trends were plotted using linear regression and annual rates of decline determined based on the linear regression slope. Differences among groups were assessed using 1-way analysis of variance. Two-tailed tests were performed, and a *P* < .05 was regarded as statistically significant. To evaluate the practical significance of the findings, Cohen *d* was calculated as an effect size measure to quantify the magnitude and practical relevance of the observed group differences. Effect sizes of 0.2 to 0.3 were considered small; 0.5, medium; and ≥0.8, large. Data analysis and visualization were performed using R version 4.5.1 (R Project for Statistical Computing) and Prism version 10.5.0 (GraphPad).

## Results

### Total Nicotine Consumption at Different Levels of Remoteness

**Figure 1.**
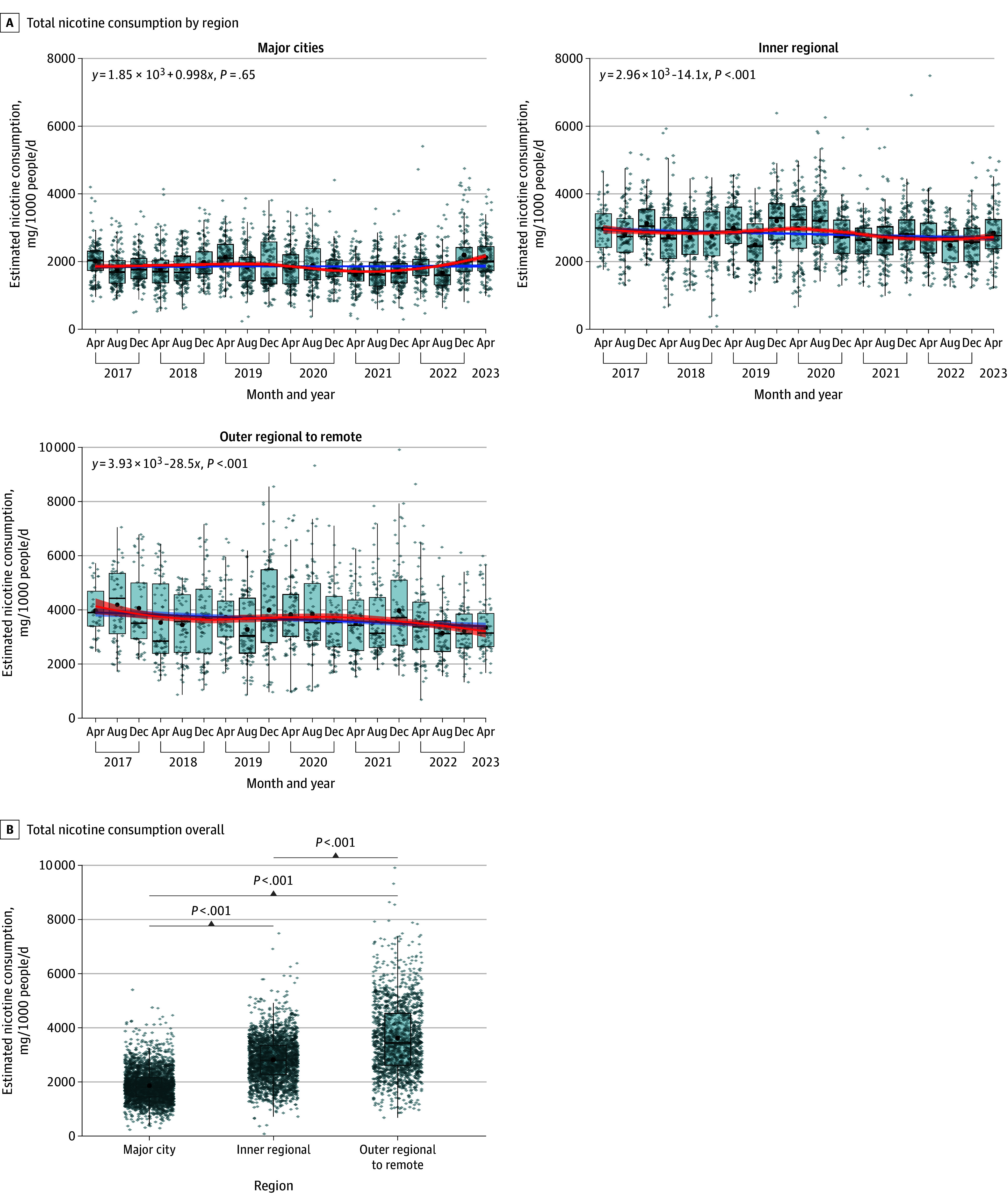
Consumption of Total Nicotine by Level of Remoteness, 2017 to 2023 The box plots display the 25th and 75th percentiles (box edges), median (center line), and 5th and 95th percentiles (whiskers). The black dots represent the mean consumption estimate in the period, and the gray diamonds represent individual measurements. The red line represents the LOESS localized polynomial smoothing, and the blue line represents the simple linear regression (with text at the top of the plot indicating the linear equation), with shaded areas presenting the 95% CIs.

In the period of 2017 to 2023, wastewater samples from as many as 55 wastewater treatment plants, covering 14 million people, were collected from 3 remoteness levels in Australia, resulting in a total of 5987 measurements. Total nicotine consumption data were retrieved from the National Wastewater Drug Monitoring Program.^23^ Temporal trends in total nicotine consumption varied over time in different remoteness categories (Figure 1A). Daily total nicotine consumption in major cities remained stable (annual change: 0.2%; 95% CI, −0.5% to 0.9%; *P* = .65) but declined significantly in inner regional (annual change: −1.4%; 95% CI, −2.1% to −0.8%; *P* < .001) and outer regional to remote (annual change: −2.2%; 95% CI, −3.2% to −1.1%; *P* < .001) areas.

Mean total nicotine consumption differed significantly by the level of remoteness across Australia (Figure 1B). Total mean (SD) nicotine consumption in major cities (1860 [602] mg/1000 people/d; 95% CI, 1840-1890 mg/1000 people/d) was significantly lower than in inner regional areas (2820 [811] mg/1000 people/d; 95% CI, 2780-2850 mg/1000 people/d) (*P* < .001; Cohen *d* = 1.36) and outer regional to remote areas (3630 [1350] mg/1000 people/d; 95% CI, 3560-3700 mg/1000 people/d) (*P* < .001; Cohen *d* = 1.88) (Figure 1B). Lastly, total nicotine consumption in inner regional areas was significantly lower than that in outer regional to remote areas (*P* < .001; Cohen *d* = 0.76).

### Tobacco-Derived Nicotine Consumption at Different Levels of Remoteness

**Figure 2.**
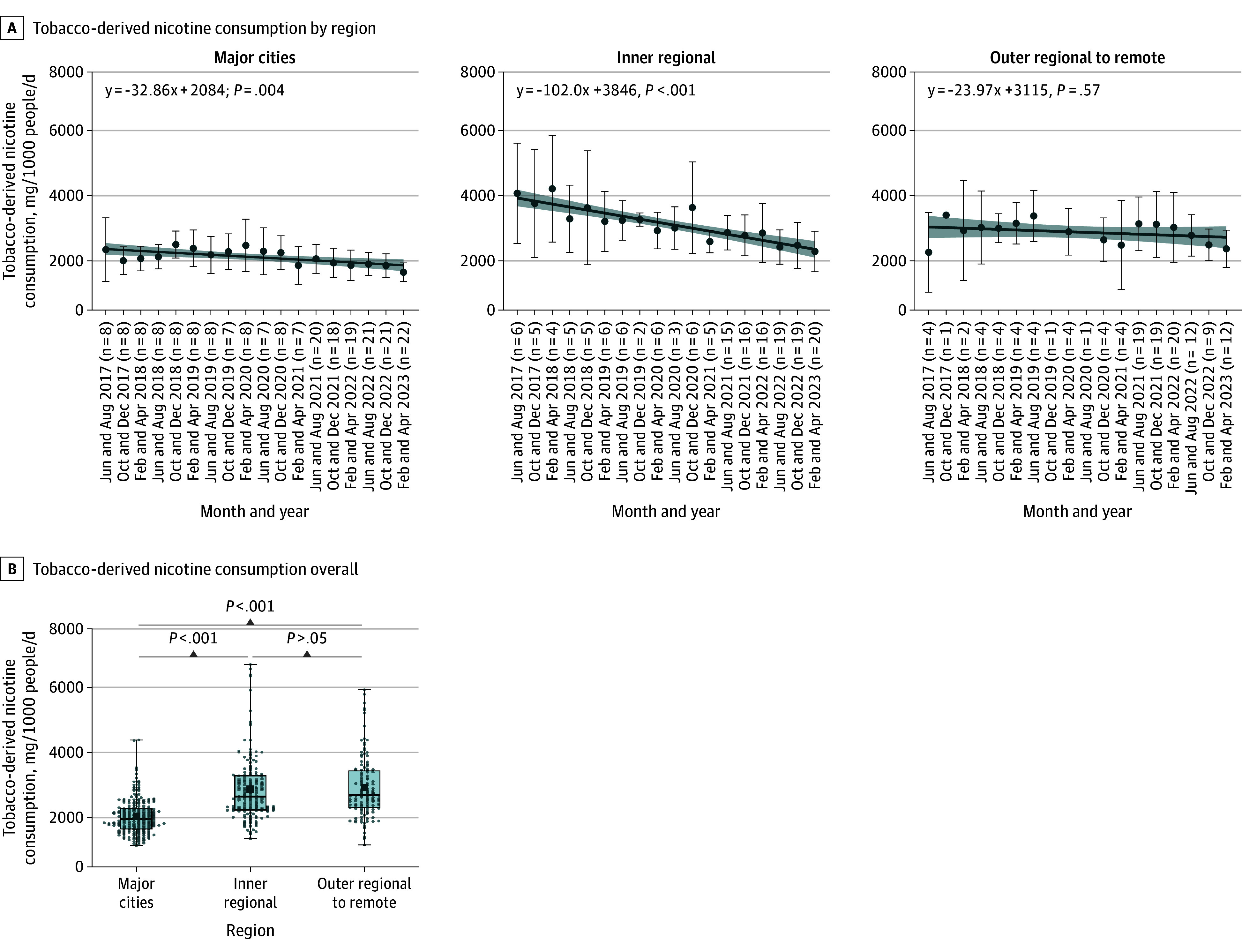
Consumption of Tobacco-Derived Nicotine by Level of Remoteness A, The dots and whiskers indicate the medians and IQRs, respectively. The lines represent simple linear regression, with the corresponding regression equation displayed at the top of the plot, and the shaded areas presenting the 95% CI. B, The box plots display the 25th and 75th percentiles (box edges), median (center line), and 5th and 95th percentiles (whiskers). The black dots represent the mean consumption estimate in the period.

Anabasine was used to track tobacco-derived nicotine consumption trends. Temporal trends in tobacco-derived nicotine consumption varied in different categories of remoteness from 2017 to 2023 (Figure 2A). Daily tobacco-derived nicotine consumption decreased significantly in both major cities (annual change: −5.0%; 95% CI, −8.3% to −1.9%; *P* = .004) and inner regional areas (annual change: −9.8%; 95% CI, −12.5% to −7.3%; *P* < .001). These decreasing rates were faster than the decline in total nicotine consumption. However, there was not a statistically significant decrease in outer regional to remote areas (annual change: −2.3%; 95% CI, −6.0% to 1.8%; *P* = .57), which showed a similar decreasing rate to the decline of total nicotine consumption.

Mean (SD) tobacco-derived nicotine consumption in major cities (1690 [580] mg/1000 people/d; 95% CI, 1610-1770 mg/1000 people/d) was significantly lower than in inner regional areas (2600 [1070] mg/1000 people/d; 95% CI, 2430-2760 mg/1000 people/d) and outer regional to remote areas (2660 [1010] mg/1000 people/d; 95% CI, 2480-2840 mg/1000 people/d) (*P* < .001) (Figure 2B). The observed Cohen *d* values are 1.03 and 1.16, suggesting substantial differences in tobacco-derived nicotine consumption between major cities and the other 2 regions. There was no difference between inner regional and outer regional to remote areas (*P* = .55, Cohen *d* = 0.06).

### National Trends for Total Nicotine and Tobacco-Derived Nicotine Consumption

**Figure 3.**
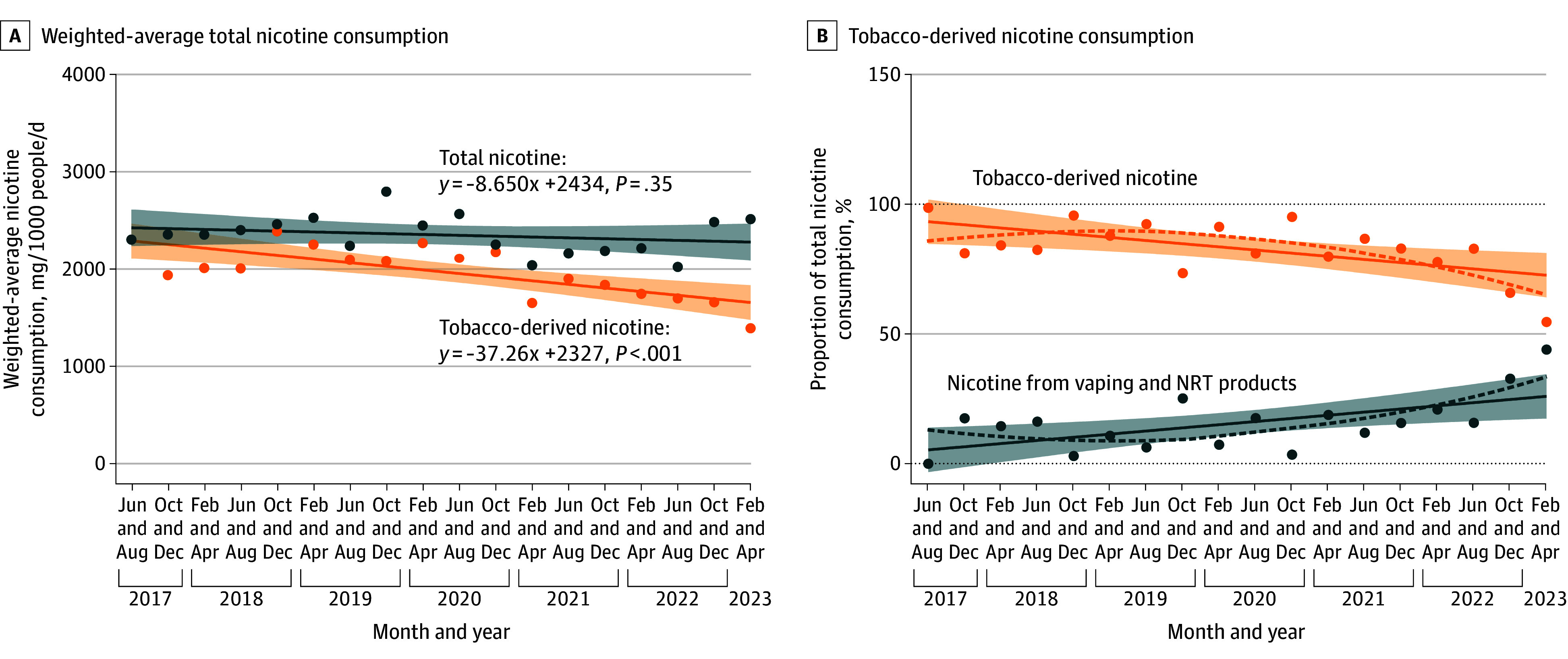
Comparison Between Weighted-Average Total Nicotine and Tobacco-Derived Nicotine Consumption, 2017 to 2023 The colored lines represent the simple linear regression, with the equation of the lines displayed at the top of the plot. The shaded areas present the 95% CI. Anabasine was not analyzed in Western Australia and South Australia samples due to limited resources, so in this figure, data were not included for total nicotine consumption.

To compare the total nicotine and tobacco-derived nicotine consumption at the national level, the national weighted-average estimates were calculated using Equation 4, and the temporal trends are presented in Figure 3A. Total nicotine consumption was stable over the studied period (*P* = .35), but tobacco-derived nicotine consumption had a significant decline of 28% (95% CI, 13%-40%; *P* < .001).

The percentage of tobacco-derived nicotine relative to total nicotine consumption was calculated (Figure 3B), with an overall decline from 94.6% (95% CI, 85.9% to 103.3%) in 2017 to 74.0% (95% CI, 65.0% to 82.4%) in 2023. Correspondingly, the proportion of nicotine consumption sourced from both vaping and nicotine replacement therapy (NRT) products increased from 5.4% (95% CI, −3.3% to 14.0%) to 26.3% (95% CI, 17.6% to 35.0%) over the same period (Figure 3B).

### Estimates of Illicit Tobacco Trends

**Figure 4.**
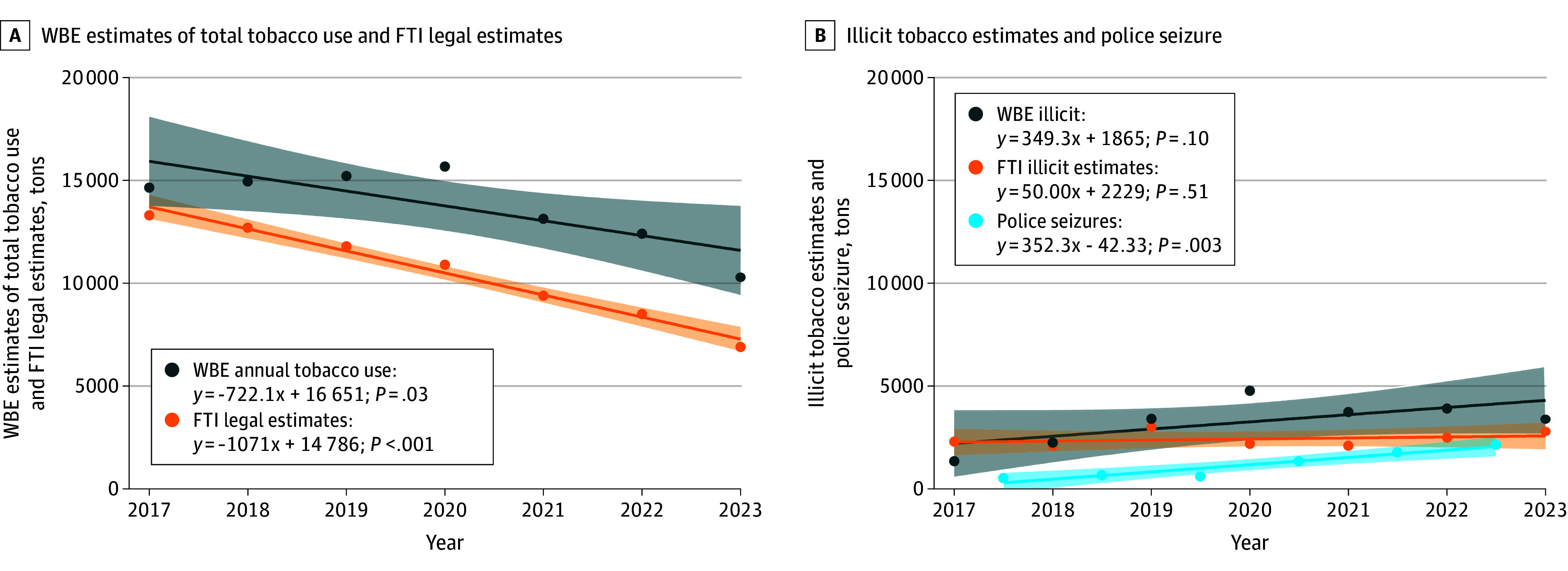
Trends in Total, Legal, and Illicit Tobacco Use Estimates A, Trends of wastewater-based estimates (WBE) of total tobacco use and FTI estimates of legal tobacco use. B, shows trends of WBE estimates and FTI estimates of illicit tobacco use. WBE estimates annual tobacco use, indicates annual tobacco use based on wastewater estimates; WBE estimates illicit, illicit tobacco estimation calculated by comparing WBE estimates annual tobacco use and FTI estimates legal. FTI estimates legal, FTI estimates illicit, and police seizures data come from the reports of Illicit tobacco in Australia, published by FTI Consulting.^28^ FTI estimates legal presents the legal sales tobacco data; FTI estimates illicit, the estimation of illicit tobacco data in Australia market; police seizures, the annual seizure data of illicit tobacco, covering the financial years 2017-2018 through 2022-2023.

The wastewater estimates of annual tobacco use were calculated following Equation 5, which witnessed a considerable decline from 14 650 to 10 300 tons in 2017 to 2023 (Figure 4A). When comparing with the total sales of legal tobacco (FTI legal estimates), WBE illicit estimates was estimated and is presented in Figure 4B. An increase from 1350 tons to 3400 tons was observed over the period. Wastewater estimates of illicit tobacco use were consistently higher than FTI estimates of illicit tobacco since 2019. Police seizures of illicit tobacco products over the period have also been increasing (Figure 4B).

## Discussion

In this study, we found that tobacco-derived nicotine showed an overall downward trend in all regions, with the fastest decline observed in inner regional areas. The decrease in tobacco use coincides with progressive increases in tobacco excise (from $0.70 to $1.24 per cigarette in 2017 and 2023, a 77.1% increase), a policy measure strongly associated with reduced smoking rates.^29,30^ This decline also coincides with survey results, which found the proportion of the smoking population who were motivated to attempt to quit or cut down due to the cost of tobacco significantly increased from 38.1% in 2007 to 56.8% in 2019.^31^ During the same time frame several other tobacco control measures, such as smoke-free laws, public health media campaigns, and cessation support services, were introduced. Overall, our data showed that tobacco use has continued to decline, which aligns with Australia’s strengthened tobacco control policies.

The geographic pattern of higher total nicotine and tobacco-derived nicotine consumption estimates in more remote (less urban) areas aligns with survey data on smoking prevalence in representative population-based surveys,^32,33^ which, in 2022 to 2023, showed daily smoking prevalence increases with remoteness, from 7% in major cities to 20% in remote and very remote areas.^30,33^

The share of total nicotine consumption derived from vaping and NRT products continued to increase over the study period (Figure 3B). The number of prescriptions for NRT products from the Pharmaceutical Benefits Scheme (PBS) peaked in 2020 and has since declined (eFigures 3 and 4 in Supplement 1). Concurrently, national survey data report a notable rise in vaping prevalence from 0.5% in 2016 to 3.5% in 2022 to 2023.^7^ Moreover, the increasing availability of vaping products from retail vape shops that specifically sell vaping products has been observed.^34,35^ Taken together, the increase in nicotine consumption sourced from both vaping and NRT products, coinciding with decrease in NRT prescribing, may suggest an increased uptake of vaping products.

Based on smoking and vaping prevalence from survey data and assuming the same daily nicotine intake, nicotine from vaping was estimated to account for approximately 25% of total nicotine consumption. This estimate aligns with our 2023 WBE, which attributed approximately 26.3% (95% CI, 17.6%-35.0%) of total nicotine to vaping sources. While the estimation considers only vaping prevalence, it excludes over-the-counter NRT products (eg, nicotine patches and gums).

Nicotine-containing vaping solutions are illegal in Australia, yet many vaping products sold as “nicotine-free” in fact contained nicotine.^36^ The rapid growth in the illicit vaping product market and use by teens and young adults may be driven by appealing flavors, lower cost relative to cigarettes, promotion on social media and by peers, and the increased convenience of disposable high nicotine vaping devices.^37,38^ This has been reflected in increasing prevalence of vaping in surveys with the highest prevalence in the 18 to 24 years (20%) and 25 to 34 years (17%) age groups by early 2023.^39^ Additionally, increases in vaping uptake have been observed during the period of rising tobacco taxes, with some adults reporting use of nicotine vaping products in place of cigarettes.^40^

The Australian government has introduced a series of policies to reduce the use of nonprescribed vaping products. The most recent reforms in 2024 further restricted legal access to nontherapeutic vaping products, removed some barriers to enforcement against illicit nontherapeutic vaping product sales, and increased the quality standards for therapeutic vaping products that can be supplied for smoking cessation via pharmacies, along with introducing flavor restrictions and stricter packaging and labeling requirements.^41,42^

Additionally, our study findings confirm that the amount of illicit tobacco smoked increased gradually before 2020, but declined from 2020 to 2022 and 2023, despite police seizure data increasing consistently from 2019.^28^ National survey data suggest a substantial increase in the proportion of the smoking population who purchased illicit tobacco between 2016 and 2022 to 2023.^43^ When calculating the percentage of the market that is illicit based on the wastewater estimates, the proportion increased from approximately 9% in 2017 to 33% in 2023. This coincides with the increasing proportion of the smoking population using illicit tobacco in the survey data and an increase in media reporting of police seizures of illicit tobacco during this period.^44^ It is notable that the overall amount of illicit tobacco that was smoked declined between 2020 and 2023 according to our estimates, which may be associated with the overall decrease in the number of people in Australia who smoked tobacco from 2.3 million in 2019 to 1.8 million in 2022 to 2023.^4^ However, the proportion of the illicit tobacco market share has remained relatively stable at approximately 30% since 2020. While the expansion of the illicit tobacco market share could slow Australia’s progress toward its goal of reducing smoking prevalence to 5.0% or less by 2030, it is reassuring that smoking prevalence continued to decline during a time of rapid growth in the illicit market. Nevertheless, additional efforts are necessary to control the expansion of the illicit tobacco market.

### Limitations

We acknowledge the following limitations of our study. First, the excretion factors of nicotine metabolites and anabasine can vary among individuals and subgroups, which may have an impact on the accuracy of estimates. Second, due to resource constraints, anabasine was not analyzed in all samples used to estimate tobacco use between 2017 and 2021. However, the analyzed samples still represented approximately 22% of the Australian population. While our method allows for estimation of the proportion of nicotine consumption attributable to vaping and NRT product sources, it cannot distinguish between nicotine derived from vaping products (or other nontherapeutic nicotine products, such as nicotine pouches) and that from medicinal NRT. A limitation of the NRT prescription from PBS is that it captures only part of total NRT use, as many NRT products are available over the counter without a prescription. Additional NRT use outside prescription record includes NRT supplied within hospitals and distributed via Quitline programs. Furthermore, prescription and over-the-counter sales data do not directly reflect actual NRT consumption, as early discontinuation is common.^45^ Another limitation is the use of legal tobacco estimates derived from a tobacco industry-funded report, which was necessary due to the limited publicly available data on tobacco sales.

## Conclusions

This study confirmed an overall decline in total nicotine and tobacco-derived nicotine consumption among Australian populations. Importantly, our data suggest that there has been an increase in nicotine consumption from vaping and NRT products and a growth in the illicit tobacco market share in Australia. These insights from wastewater-based epidemiology improve our understanding of the trends in the use of tobacco, vaping and NRT products and are pivotal in evaluating the effectives of policies to control the use of tobacco and other nicotine products.

## Notes

Supplement 1.
eMethods.
**eTable 1.** NWDMP Data Sample Collected Information in 2017-2023 **eTable 2.** Sample Information for Anabasine Analysis from 2017 to 2023 **eTable 3.** Results of Quality Assurance/Quality Control **eFigure 1.** Flowchart of the Back-Estimations in the Article Using Different Equations **eFigure 2.** Definitions And Interrelationships Among the Terms of Different Product Use and Different Sources of Nicotine Consumption **eFigure 3.** Number of Prescriptions (Prescriptions/Month) for Nicotine Replacement Therapy (NRT) in Australia from 2017 to 2025 **eFigure 4.** Number of Prescriptions (Prescriptions/Year) for Different Types of NRT Products in Australia Since 2008
eReference.

Supplement 2.
Data Sharing Statement
