# Supplementary material for: National Wastewater Surveillance of Illicit Tobacco and Vaping Use Trends in Australia
Source: JAMA Netw Open. 2026 Feb 9;9(2):e2557319. doi: 10.1001/jamanetworkopen.2025.57319 (PMC12887742; doi:10.1001/jamanetworkopen.2025.57319)
Supplement: Supplement 1. — eMethods. eTable 1. NWDMP Data Sample Collected Information in 2017-2023 eTable 2. Sample Information for Anabasine Analysis from 2017 to 2023 eTable 3. Results of Quality Assurance/Quality Control eFigure 1. Flowchart of the Back-Estimations in the Article Using Different Equations eFigure 2. Definitions And Interrelationships Among the Terms of Different Product Use and Different Sources of Nicotine Consumption eFigure 3. Number of Prescriptions (Prescriptions/Month) for Nicotine Replacement Therapy (NRT) in Australia from 2017 to 2025 eFigure 4. Number of Prescriptions (Prescriptions/Year) for Different Types of NRT Products in Australia Since 2008 eReference. [file jamanetwopen-e2557319-s001.pdf]

# Supplemental Online Content

Wang Z, Zheng Q, Thai PK, et al. National wastewater surveillance of illicit tobacco and vaping use trends in Australia. *JAMA Netw Open.* 2026;9(2):e2557319. doi:10.1001/jamanetworkopen.2025.57319

**eMethods.**

**eTable 1.** NWDMP Data Sample Collected Information in 2017-2023

**eTable 2.** Sample Information for Anabasine Analysis from 2017 to 2023

**eTable 3.** Results of Quality Assurance/Quality Control

**eFigure 1.** Flowchart of the Back-Estimations in the Article Using Different Equations

**eFigure 2.** Definitions And Interrelationships Among the Terms of Different Product Use and Different Sources of Nicotine Consumption

**eFigure 3.** Number of Prescriptions (Prescriptions/Month) for Nicotine Replacement Therapy (NRT) in Australia from 2017 to 2025

**eFigure 4.** Number of Prescriptions (Prescriptions/Year) for Different Types of NRT Products in Australia Since 2008

**eReference.**

This supplemental material has been provided by the authors to give readers additional information about their work.

© 2026 Wang Z et al. *JAMA Network Open*.

## eMethods

### e2.1 Sample collection and transportation.

The wastewater samples analyzed in this study were collected by the Australian National Wastewater Drug Monitoring Program (NWDMP) from April 2017 to April 2023. One-week 24-hour composite influent wastewater samples were collected using either time-based or flow-proportional sampling methods by auto-sampler from up to 55 WWTPs across Australia. After collection, samples were preserved at pH 2 by adding 2 M HCl (1 mL per 100 mL sample) and then archived at −20 °C . Subsequently, the samples were transported frozen to The University of Queensland, where they were stored at −20 °C in dark conditions until analysis.

### e2.2 Catchment data collection.

Catchment data, including sampling information and flow data corresponding to the sampling period were provided by each respective WWTP in a sampling questionnaire. Daily flows were obtained from the WWTP to normalize each concentration for dilution. In addition, catchment boundary maps were provided by WWTPs in 2016 and 2021 as GIS or PDF files, and georeferenced as necessary. Catchment populations (usual residency) were determined by overlaying the catchment boundary maps with the smallest unit of the Australian census in 2016 and 2021 (mesh block). The intersecting areas were summed in each catchment. Where units were split between inside and outside the catchment boundary, the intersected units were weighted for population dispersion using the number of addresses in or outside the catchment boundary, the Georeferenced National Address File (GNAF), and included in the total population sum. The resulting 2016 and 2021 catchment populations were linearly interpolated to monthly, based on the change over time. The proportion of census respondents outside of their catchment on census day was determined and this was averaged 4.4% [1].

© 2026 Wang Z et al. *JAMA Network Open*.

## eTable 1. NWDMP Data Sample Collected Information in 2017-2023

| Site No. | States | Population range | Remote level |
|---|---|---|---|
| 1 | Victoria | ≥ 1,000,000 | Major Cities |
| 2 | Queensland | 200,000 - 1,000,000 | Major Cities |
| 3 | New South Wales | ≥ 1,000,000 | Major Cities |
| 4 | Tasmania | 20,000 - 50,000 | Inner Regional |
| 5 | Queensland | 200,000 - 1,000,000 | Major Cities |
| 6 | New South Wales | ≥ 1,000,000 | Major Cities |
| 7 | South Australia | 200,000 - 1,000,000 | Major Cities |
| 8 | New South Wales | 200,000 - 1,000,000 | Major Cities |
| 9 | Australian Capital Territory | 200,000 - 1,000,000 | Major Cities |
| 10 | Northern Territory | 20,000 - 50,000 | Outer Regional to Remote |
| 11 | Queensland | 200,000 - 1,000,000 | Major Cities |
| 12 | Queensland | 200,000 - 1,000,000 | Major Cities |
| 13 | South Australia | 200,000 - 1,000,000 | Major Cities |
| 14 | New South Wales | 20,000 - 50,000 | Inner Regional |
| 15 | South Australia | < 20,000 | Outer Regional to Remote |
| 16 | Tasmania | 20,000 - 50,000 | Inner Regional |
| 17 | Tasmania | 20,000 - 50,000 | Inner Regional |

© 2026 Wang Z et al. *JAMA Network Open*.

| Site No. | States | Population range | Remote level |
|---|---|---|---|
| 18 | South Australia | < 20,000 | Inner Regional |
| 19 | Queensland | 20,000 - 50,000 | Outer Regional to Remote |
| 20 | New South Wales | 50,000 - 200,000 | Major Cities |
| 21 | South Australia | 50,000 - 200,000 | Major Cities |
| 22 | Queensland | 50,000 - 200,000 | Outer Regional to Remote |
| 23 | Queensland | 50,000 - 200,000 | Inner Regional |
| 24 | Queensland | 50,000 - 200,000 | Inner Regional |
| 25 | Victoria | 200,000 - 1,000,000 | Inner Regional |
| 26 | Tasmania | < 20,000 | Inner Regional |
| 27 | Tasmania | < 20,000 | Outer Regional to Remote |
| 28 | Queensland | 20,000 - 50,000 | Inner Regional |
| 29 | South Australia | 50,000 - 200,000 | Major Cities |
| 30 | Victoria | 50,000 - 200,000 | Inner Regional |
| 31 | South Australia | 20,000 - 50,000 | Outer Regional to Remote |
| 32 | Victoria | 20,000 - 50,000 | Inner Regional |
| 33 | Victoria | ≥ 1,000,000 | Major Cities |
| 34 | New South Wales | 200,000 - 1,000,000 | Major Cities |
| 35 | South Australia | < 20,000 | Inner Regional |
| 36 | Queensland | < 20,000 | Outer Regional to Remote |
| 37 | Northern Territory | 20,000 - 50,000 | Outer Regional to Remote |
| 38 | New South Wales | < 20,000 | Outer Regional to Remote |
| 39 | Western Australia | 200,000 - 1,000,000 | Major Cities |
| 40 | Western Australia | 50,000 - 200,000 | Inner Regional |
| 41 | Western Australia | 200,000 - 1,000,000 | Major Cities |
| 42 | Western Australia | 200,000 - 1,000,000 | Major Cities |
| 43 | Victoria | 50,000 - 200,000 | Inner Regional |
| 44 | New South Wales | 20,000 - 50,000 | Inner Regional |
| 45 | South Australia | < 20,000 | Outer Regional to Remote |
| 46 | Western Australia | 20,000 - 50,000 | Outer Regional to Remote |
| 47 | Victoria | < 20,000 | Outer Regional to Remote |
| 48 | Victoria | < 20,000 | Inner Regional |
| 49 | Victoria | 20,000 - 50,000 | Inner Regional |
| 50 | Western Australia | 20,000 - 50,000 | Outer Regional to Remote |
| 51 | Victoria | 20,000 - 50,000 | Inner Regional |
| 52 | Victoria | 20,000 - 50,000 | Inner Regional |
| 53 | New South Wales | < 20,000 | Inner Regional |
| 54 | New South Wales | < 20,000 | Inner Regional |
| 55 | New South Wales | < 20,000 | Outer Regional to Remote |

Note: One-week daily wastewater samples were collected and analysed every four months.

© 2026 Wang Z et al. *JAMA Network Open*.

## eTable 2. Sample Information for Anabasine Analysis from 2017 to 2023

**First Batch: Jun 2017 - Jun 2023**

| Site No. | States | Population | Remoteness |
|---|---|---|---|
| 2 | Queensland | 200,000 - 1,000,000 | Major Cities |
| 3 | New South Wales | ≥ 1,000,000 | Major Cities |
| 4 | Tasmania | 20,000 - 50,000 | Inner Regional |
| 5 | Queensland | 200,000 - 1,000,000 | Major Cities |
| 6 | New South Wales | ≥ 1,000,000 | Major Cities |
| 8 | New South Wales | 200,000 - 1,000,000 | Major Cities |
| 9 | Australian Capital Territory | 200,000 - 1,000,000 | Major Cities |
| 10 | Northern Territory | 20,000 - 50,000 | Outer Regional to Remote |
| 12 | Queensland | 200,000 - 1,000,000 | Major Cities |
| 14 | New South Wales | 20,000 - 50,000 | Inner Regional |
| 17 | Tasmania | 20,000 - 50,000 | Inner Regional |
| 22 | Queensland | 50,000 - 200,000 | Outer Regional to Remote |
| 24 | Queensland | 50,000 - 200,000 | Inner Regional |
| 25 | Victoria | 200,000 - 1,000,000 | Inner Regional |
| 34 | New South Wales | 200,000 - 1,000,000 | Major Cities |
| 47 | Victoria | < 20,000 | Outer Regional to Remote |
| 48 | Victoria | < 20,000 | Inner Regional |
| 49 | Victoria | 20,000 - 50,000 | Inner Regional |
| Note: One-week daily wastewater samples of every two months were aliquoted and pooled into one sample. | | | |

**Second Batch: Jun 2021 - Apr 2023**

| Site No. | States | Population | Remoteness |
|---|---|---|---|
| 1 | Victoria | ≥ 1,000,000 | Major Cities |
| 7 | South Australia | 200,000 - 1,000,000 | Major Cities |
| 11 | Queensland | 200,000 - 1,000,000 | Major Cities |
| 13 | South Australia | 200,000 - 1,000,000 | Major Cities |
| 15 | South Australia | < 20,000 | Outer Regional to Remote |
| 16 | Tasmania | 20,000 - 50,000 | Inner Regional |
| 18 | South Australia | < 20,000 | Inner Regional |
| 19 | Queensland | 20,000 - 50,000 | Outer Regional to Remote |
| 20 | New South Wales | 50,000 - 200,000 | Major Cities |
| 21 | South Australia | 50,000 - 200,000 | Major Cities |
| 23 | Queensland | 50,000 - 200,000 | Inner Regional |
| 26 | Tasmania | < 20,000 | Inner Regional |
| 27 | Tasmania | < 20,000 | Outer Regional to Remote |
| 28 | Queensland | 20,000 - 50,000 | Inner Regional |
| 29 | South Australia | 50,000 - 200,000 | Major Cities |

© 2026 Wang Z et al. *JAMA Network Open*.

| | | | |
|---|---|---|---|
| 30 | Victoria | 50,000 - 200,000 | Inner Regional |
| **Site No.** | **States** | **Population** | **Remoteness** |
| 31 | South Australia | 20,000 - 50,000 | Outer Regional to Remote |
| 32 | Victoria | 20,000 - 50,000 | Inner Regional |
| 33 | New South Wales | ≥ 1,000,000 | Major Cities |
| 35 | South Australia | < 20,000 | Inner Regional |
| 36 | Queensland | < 20,000 | Outer Regional to Remote |
| 37 | Northern Territory | 20,000 - 50,000 | Outer Regional to Remote |
| 38 | New South Wales | < 20,000 | Outer Regional to Remote |
| 39 | Western Australia | 200,000 - 1,000,000 | Major Cities |
| 40 | Western Australia | 50,000 - 200,000 | Inner Regional |
| 41 | Western Australia | 200,000 - 1,000,000 | Major Cities |
| 42 | Western Australia | 200,000 - 1,000,000 | Major Cities |
| 43 | Victoria | 50,000 - 200,000 | Inner Regional |
| 44 | New South Wales | 20,000 - 50,000 | Inner Regional |
| 45 | South Australia | < 20,000 | Outer Regional to Remote |
| 46 | Western Australia | 20,000 - 50,000 | Outer Regional to Remote |
| 50 | Western Australia | 20,000 - 50,000 | Outer Regional to Remote |
| 51 | Victoria | 20,000 - 50,000 | Inner Regional |
| 52 | Victoria | 20,000 - 50,000 | Inner Regional |
| 53 | New South Wales | < 20,000 | Inner Regional |
| 54 | New South Wales | < 20,000 | Inner Regional |
| 55 | New South Wales | < 20,000 | Outer Regional to Remote |

Note: One-week daily wastewater samples of every month were aliquoted and pooled into one sample.

© 2026 Wang Z et al. *JAMA Network Open*.

## eTable 3. Results of Quality Assurance/Quality Control

| Quality Assurance / Quality Control | Analysed value (ng/L, Mean ± SD) | | | |
|---|---|---|---|---|
| | Anabasine | Cotinine | Hydroxycotinine | Frequency of analysis |
| Procedural blank | n.d. | n.d. | n.d. | Twice per batch |
| Non-extracted side spike (NESS) at 1000 ng/L | 1038 ± 83 | 919 ± 22 | 925 ± 46 | Twice per batch |
| Milli-Q blank | n.d. | n.d. | n.d. | Every 20 samples |
| Native standard at 10 ng/L | 10 ± 0.76 | 10 ± 0.83 | 11 ± 1.6 | Every 20 samples |
| Native standard at 100 ng/L | 102 ± 11 | 105 ± 2.9 | 107 ± 2.5 | Every 20 samples |
| | Accuracy (%) | | | |
| Duplicate Wastewater Samples (DUP) | 100 ± 3.7 | 98 ± 3.2 | 99 ± 3.0 | Every 10 samples |
| Spiked Wastewater (SPK) + Native 1000 ng/L | 96 ± 3.7 | 100 ± 7.3 | 105 ± 8.2 | Every 20 samples |

*Note: n.d.= not detected.

Accuracy of DUP = Conc DUP / = Conc Non-spiked Wastewater * 100%

Accuracy of SPK = (Conc SPK - Conc Non-spiked Wastewater) / Conc NESS *100%

© 2026 Wang Z et al. *JAMA Network Open*.

## eFigure 1. Flowchart of the Back-Estimations in the Article Using Different Equations

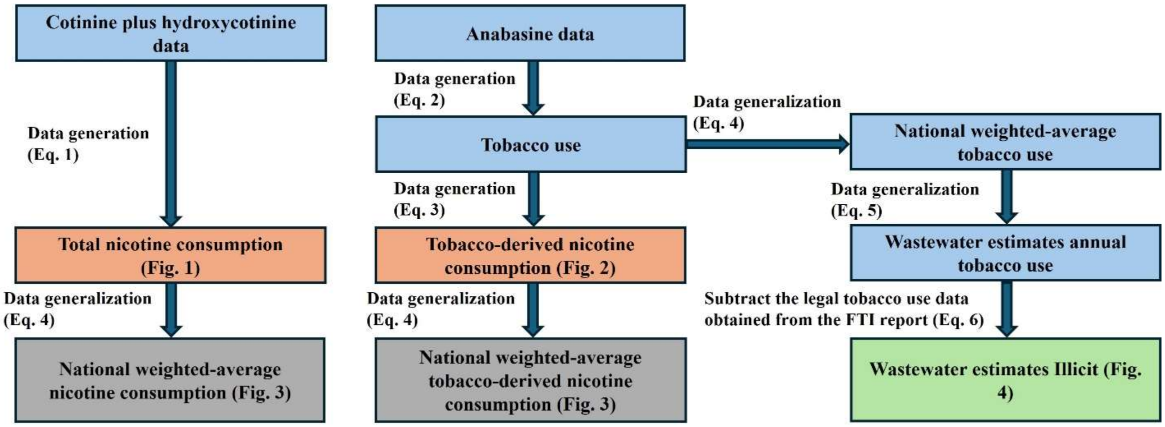


© 2026 Wang Z et al. *JAMA Network Open*.

**eFigure 2. Definitions And Interrelationships Among the Terms of Different Product Use and Different Sources of Nicotine Consumption**

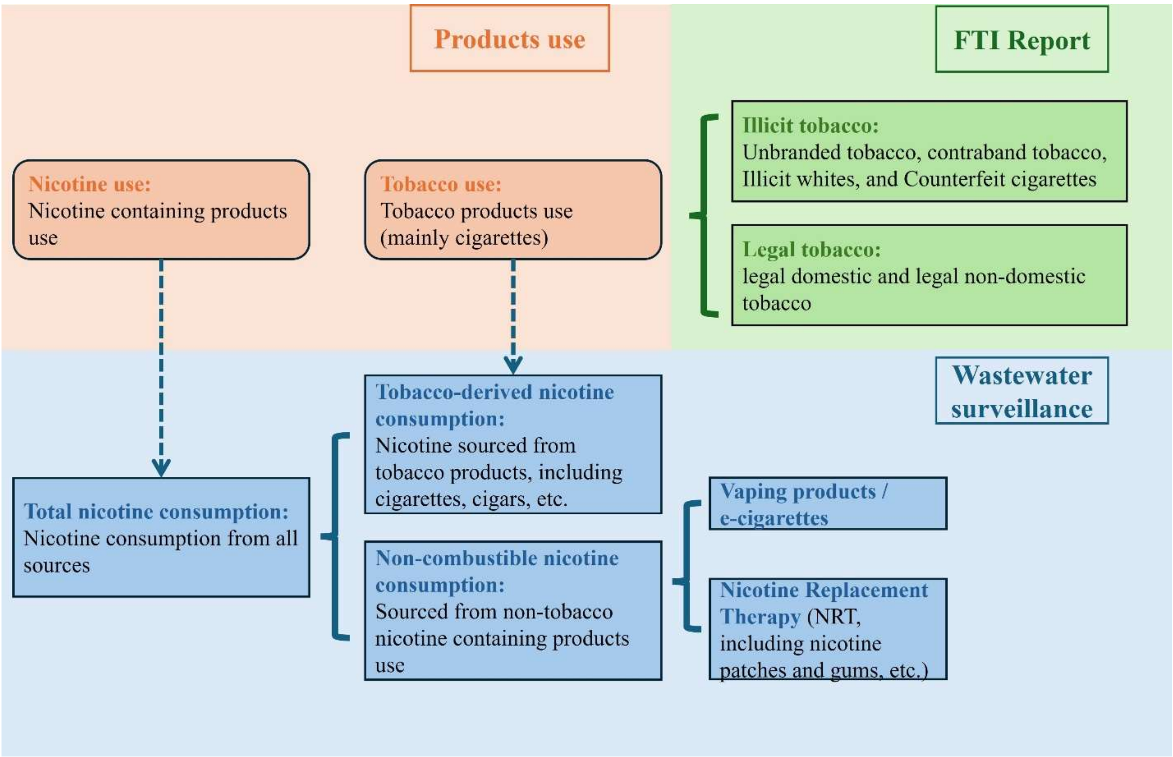


© 2026 Wang Z et al. *JAMA Network Open*.

**eFigure 3. Number of Prescriptions (Prescriptions/Month) for Nicotine Replacement Therapy (NRT) in Australia from 2017 to 2025**

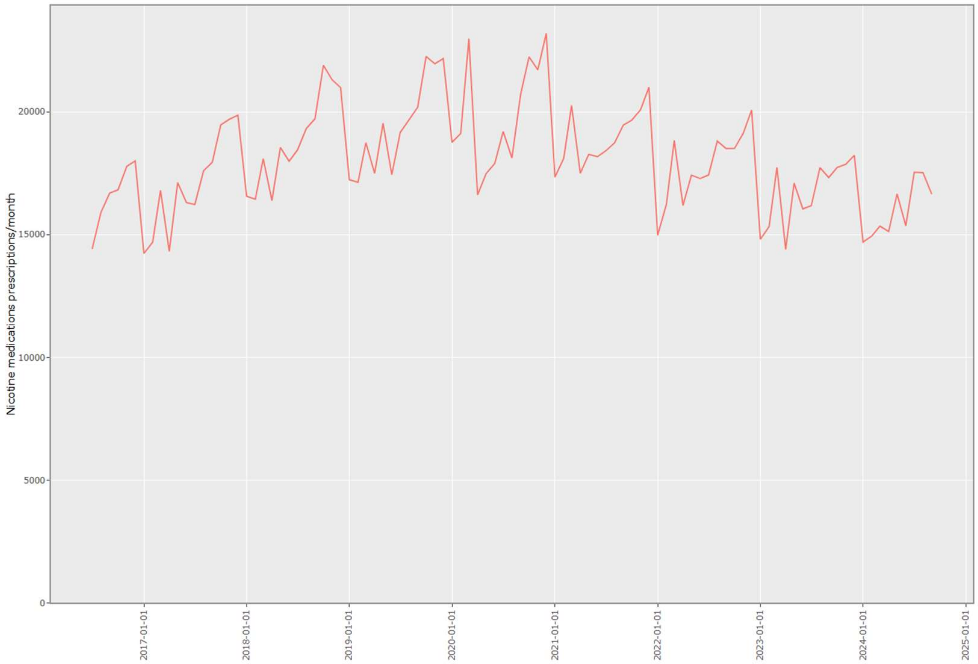


© 2026 Wang Z et al. *JAMA Network Open*.

**eFigure 4. Number of Prescriptions (Prescriptions/Year) for Different Types of NRT Products in Australia Since 2008**

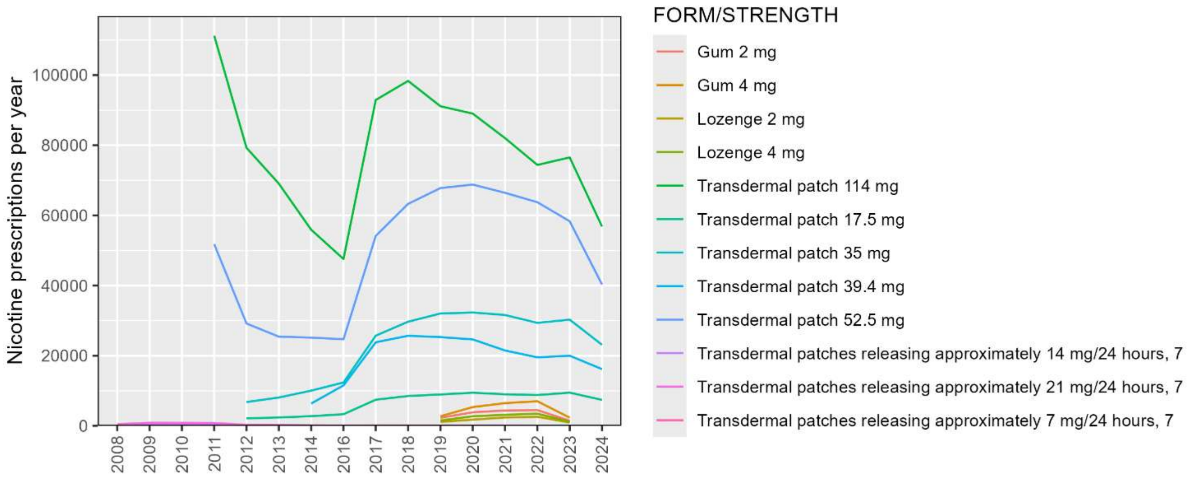


© 2026 Wang Z et al. *JAMA Network Open*.

**eReference.**

1. Tscharke, B.J., J.W. O'Brien, C. Ort, et al. Harnessing the Power of the Census: Characterizing Wastewater Treatment Plant Catchment Populations for Wastewater-Based Epidemiology. Environmental Science & Technology. 2019. **53**(17): p. 10303-10311.

© 2026 Wang Z et al. *JAMA Network Open*.
