# Supplementary material for: National Wastewater Surveillance of Illicit Tobacco and Vaping Use Trends in Australia
Source: JAMA Netw Open. 2026 Feb 9;9(2):e2557319. doi: 10.1001/jamanetworkopen.2025.57319 (PMC12887742; doi:10.1001/jamanetworkopen.2025.57319)
Supplement: Supplement 2. — Data Sharing Statement [file jamanetwopen-e2557319-s002.pdf]

Data Sharing Statement

Wang. National Wastewater Surveillance of Illicit Tobacco and Vaping Use Trends in Australia. *JAMA Netw Open*. Published February 04, 2026. doi:10.1001/jamanetworkopen.2025.57319

Data
Data available: Yes
Data types: Data (not involving human participants)
How to access data: Data (deidentified) will be available upon request
When available: With publication

Supporting Documents
Document types: None

Additional Information
Who can access the data: researchers whose proposed use of the data has been approved
Types of analyses: for research purpose
Mechanisms of data availability: with investigator support
